# A systematic review of genetic mutations in pulmonary arterial hypertension

**DOI:** 10.1186/s12881-017-0440-5

**Published:** 2017-08-02

**Authors:** Gerardo Garcia-Rivas, Carlos Jerjes-Sánchez, David Rodriguez, José Garcia-Pelaez, Victor Trevino

**Affiliations:** 1 Cátedra de Cardiología y Medicina Vascular, Escuela de Medicina y Ciencias de la Salud, Tecnologico de Monterrey, Monterrey, Mexico; 2 Centro de Investigación Biomédica, Hospital Zambrano-Hellion, Tec Salud, Tecnologico de Monterrey, San Pedro Garza García, Mexico; 3 Cátedra de Bioinformática, Escuela de Medicina y Ciencias de la Salud, Tecnologico de Monterrey, Av Morones Prieto No. 3000 Colonia Los Doctores, 64710 Monterrey, Nuevo León Mexico

**Keywords:** Genetic panel, Germ-line mutation, Heterozygote detection, Systematic review

## Abstract

**Background:**

Pulmonary arterial hypertension (PAH) is a group of vascular diseases that produce right ventricular dysfunction, heart failure syndrome, and death. Although the majority of patients appear idiopathic, accumulated research work combined with current sequencing technology show that many gene variants could be an important component of the disease. However, current guidelines, clinical practices, and available gene panels focus the diagnosis of PAH on a relatively low number of genes and variants associated with the bone morphogenic proteins and transforming Growth Factor-β pathways, such as the *BMPR2*, *ACVRL1*, *CAV1*, *ENG*, and *SMAD9*.

**Methods:**

To provide an expanded view of the genes and variants associated with PAH, we performed a systematic literature review. Facilitated by a web tool, we classified, curated, and annotated most of the genes and PubMed abstracts related to PAH, in which many of the mutations and variants were not annotated in public databases such as ClinVar from NCBI. The gene list generated was compared with other available tests.

**Results:**

Our results reveal that there is genetic evidence for at least 30 genes, of which 21 genes shown specific mutations. Most of the genes are not covered by current available genetic panels. Many of these variants were not annotated in the ClinVar database and a mapping of these mutations suggest that next generation sequencing is needed to cover all mutations found in PAH or related diseases. A pathway analysis of these genes indicated that, in addition to the BMP and TGFβ pathways, there was connections with the nitric oxide, prostaglandin, and calcium homeostasis signalling, which may be important components in PAH.

**Conclusion:**

Our systematic review proposes an expanded gene panel for more accurate characterization of the genetic incidence and risk in PAH. Their usage would increase the knowledge of PAH in terms of genetic counseling, early diagnosis, and potential prognosis of the disease.

**Electronic supplementary material:**

The online version of this article (doi:10.1186/s12881-017-0440-5) contains supplementary material, which is available to authorized users.

## Background

Pulmonary arterial hypertension (PAH) is a group of vascular diseases that are characterized by a progressive increase in pulmonary vascular resistance and pulmonary arterial pressure with secondary vascular and right ventricular remodeling. PAH leads to right ventricular dysfunction, heart failure syndrome, and finally, premature death [1]. PAH is characterized hemodynamically by the presence of pre-capillary pulmonary hypertension as defined by a pulmonary arterial wedge ≤15 mmHg and a pulmonary vascular resistance >3 Wood units in the absence of other causes of pre-capillary pulmonary hypertension. The current PAH Group 1 classifications include idiopathic, heritable, drug- and toxin-induced, and associated with connective tissue disease, human immunodeficiency virus infection, portal hypertension, congenital heart disease, and schistosomiasis [2]. Despite current therapeutic options, PAH remains a catastrophic disease that is related to an unacceptable high mortality [3]. It is clinically silent in early stages; symptoms develop late in the course of the disease. Although early diagnosis is associated with improved long-term survival, at present, most patients are diagnosed at a very advanced stage of PAH indicating that the early screening for PAH is crucial [4]. In this regard, genetic testing is an effective strategy for the early diagnosis and management of PAH. The availability of molecular diagnosis has opened up a new field in patient care, which includes genetic counseling for severe diseases; however, the major predisposing gene for PAH, BMPR2, has a highly variable penetrance within families [5]. Physicians who manage PAH should understand the heritable PAH phenotypes and the genes that are potentially responsible for PAH. They also should know the potential benefits, and issues surrounding genetic counseling and testing for patients with PAH.

The main genetic cause of familial PAH is caused by mutations in the gene bone morphogenic protein receptor type 2 (*BMPR2*) [5]. Although mutations in specific protein domains have been studied [6], over 300 different *BMPR2* mutations along the gene have been related to the diagnosis and prognosis of PAH [5, 7]. Recent studies have suggested that a BMPR2 mutation promotes cell division and prevents cell death, resulting in an overgrowth of cells in the small arteries throughout the lungs [8]. As a result, these arteries narrow in diameter, which increases the resistance to blood flow and, consequently, increases the end-diastolic volume and causes adverse right ventricular remodeling. In addition, new evidence suggests that, in spite of a similar afterload, the right ventricular function is more severely affected in *BMPR2*-mutation carriers that in noncarriers [9]. Although mutations in the *BMPR2* gene are the single most common causal factor for hereditary cases of PAH, other pathogenic mutations have been observed in approximately 25% of idiopathic PAH and the occurrence of novel mutations in patients with PAH who have a family history can be 20–30% [5]. Recent progress in the development of next-generation sequencing has facilitated the evaluation of several novel mutations that strongly correlate with PAH [10]. The current genetic testing panels for PAH-associated autosomal dominant genes available mainly test for BMPR2-related genes; novel and not frequently represented genes are not included.

In this systematic review, we present a classification of the evidence of genetic mutations for PAH that are reported in the literature. These results can be used to suggest a comprehensive gene panel that is implicated in PAH for screening purposes. Additionally, this systematic classification avoids biases or overrepresentation of well-known genes, it is retrospective, depends on primary sources, and considers information from diverse cohorts and unrepresented minority populations of PAH patients. We also provide a functional analysis of the involved genes whose results might provide interesting insights into etiology and progression of the disease. To our knowledge, this is the first systematic review of genetic mutations in PAH.

## Methods

**Fig. 1:**
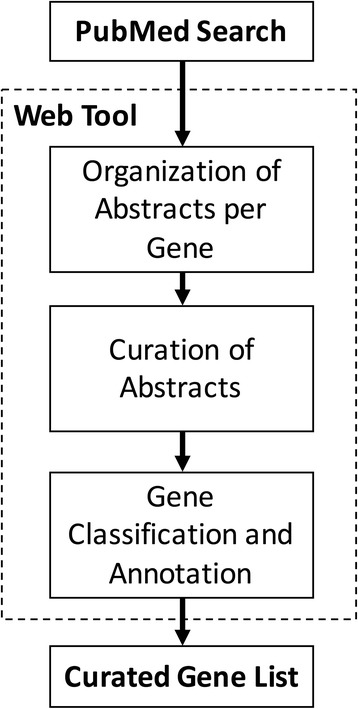
A schematic overview of the systematic review

An overview of the methodology is schematized in Fig. 1. Briefly, we collected abstracts related to mutations in PAH from PubMed. Each abstract was systematically annotated for gene names and symbols. The abstracts were computationally organized by genes to be manually revised. After the revision, the genes were classified to determine which genes showed evidence of mutations in PAH. The following sections will provide the details of this protocol.

### Search strategy

To unbiasedly obtain the most updated information on mutated genes, we acquired PubMed abstracts related to reported polymorphisms in PAH queried in May 2015. We used the following query terms: “(pulmonary arterial hypertension [TIAB]) AND (mutations [TIAB] OR mutation [TIAB] OR polymorphisms [TIAB] OR polymorphism [TIAB]).” The analysis was restricted to abstracts published in English. We used a web tool developed in our group called Pub-Term (http://bioinformatica.mty.itesm.mx:8080/Biomatec/pubterm.html) to organize, annotate, and curate abstracts per gene.

### Web tool

We used the PubTerm tool to revise, annotate, and classify genes according to the evidence of mutations in PAH. The PubTerm tool developed in our research group used the PubMed programmatic interface (http://www.ncbi.nlm.nih.gov/home/develop/api.shtml) to acquire abstracts for a given query. PubTator [11] was used within the PubTerm tool to annotate abstracts associated with genes, species, diseases, and chemicals. The PubTerm tool organizes abstracts by genes, chemicals, diseases, species, and other terms, or by the co-occurrences of genes and diseases that facilitate the classification, annotation, curation, and tracking of relevant genes. The terms within the text abstract could be highlighted to facilitate annotations. The abstracts, genes, or other terms were annotated and categorized. Each PubTerm session could be saved and retrieved afterward. PubTerm also provided statistics on the number of abstracts per gene or term, as well as the estimations of the likelihood that a given term (e. g. gene) can be found by chance in the set of retrieved abstracts.

### The organization, annotation, and curation of abstracts per gene

We used the option “Term View” within PubTerm to revise the abstracts per gene. The abstracts per gene were manually reviewed and the corresponding gene was categorized by level of importance into: *(i) annotation error, (ii) unrelated, (iii) negative evidence, (iv) related but not mutated, (v) mutation evidence in PAH, (vi) other genetic alterations in PAH,* and *(vii) genetic evidence in PAH-related disease*. The details of these criteria are shown in Table 1. Full-text reviews were made if polymorphisms or mutations in a gene were not reported in ClinVar or there was no clear evidence of mutations in the abstract.

**Table 1:** Level of evidence for gene mutations

Level	Criteria
Annotation error	When the record is related to a different disease or biological topic (mainly due to imprecisions in the gene and disease systematic annotations)
Unrelated	When the record is properly annotated but the mention of disease or the gene is casual or not the main topic
Negative evidence	When studies test gene polymorphisms and dismiss a relationship with PAH
Biologically related but no evidence of mutation	When the studies performed experiments in non-human specimens or cell cultures showing a biological relationship with PAH, treatment or development of the disease but not evidence of mutations in patients is mentioned
Experimental evidence of mutations	When experimental evidence of mutation is shown in a group of patients
Other evidences of genetic alterations	When not specific gene mutations are provided such as haplotypes or GWAS (genome wide association studies)
Genetic alteration in a related disease	When experimental evidence of mutations is provided not specifically for PAH or PAH is not the main topic

### Gene revision

For genes that occurred in the abstracts more than 10 times, we revised the abstracts in descending order by date until a definitive relation with PAH was found (typically after reading around five abstracts) or a clear annotation error was determined. For genes that occurred in less than 10 abstracts, in which their possible relationship with PAH was not clear, a manual review of all related abstracts was performed to identify a potential relationship. Only human genes were reviewed, which were filtered by using “Hs.” for *Homo sapiens* within the term view in PubTerm. The definitive sentences in the abstract were marked and included in the notes per gene. The annotations could be revised and retrieved in the PubTerm tool (using the restore session option in http://bioinformatica.mty.itesm.mx:8080/Biomatec/pubterm.html and the field values ID = *BMC-Med-Gen*, e-Mail=vtrevino@itesm.mx, Project = *PAH-Gene-Panel-405*).

### Study eligibility

All investigators designed the search. One investigator acquired the data (DR) in PubTerm and the initial annotations were done by two investigators (DR and JGP). The annotations of the revised genes were further reviewed by two more investigators (VT and GGR). Any disagreements were resolved by discussion (DR, JGP, VT, GGR, and CJ). No attempts were made to contact the authors of the original articles for information. Investigators were not blinded to the journal, author, or institutions.

### Gene model charts

We used the NCBI recommended reference mRNA sequence annotation and the isoform 1 to obtain exon counts and positions. For well-known PAH genes, we used ClinVar annotations [12] to mark and annotate the positions of the mutations. Only small variants (mutations, insertions, and deletions) and those not benign nor likely benign were used. Only variants annotated to the reference mRNA were used. For genes with mutations that were not reported in ClinVar, the information of the mutation was obtained from the abstract or the full text or the article. In these cases, the records are identified with the title “Manually annotated”. The list and details (such as positions and citation) of mutations generated are shown in Additional file 1.

### Functional analysis

We used DAVID [13] and EnrichR [14] to functionally annotate the generated list of genes that had *experimental evidence of mutations* or *other evidence of genetic alterations* (21 and 9 genes, respectively). DAVID/EnrichR performs an over-representation test using databases of biological terms listing the terms that significantly overlap with the list of genes. From the generated lists of biological terms, we grouped terms based on the similarity of gene content using hierarchical clustering where the resulted clusters were clearly associated with a consensus topic. This analysis was performed separately for terms related to Gene Ontology (GO) and non-GO terms commonly related to pathways.

## Results

### Classification and identification of PAH genes

**Fig. 2:**
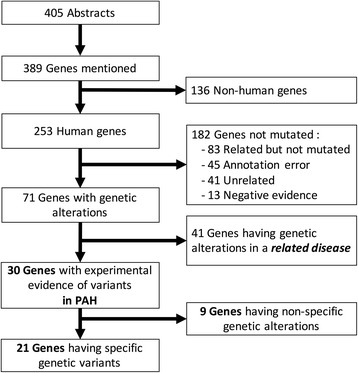
Results of the curation process from abstracts to genes

From 2004 to 2015, our electronic search identified 405 abstracts involving 389 different genes mentioned in the query for PAH mutations. Figure 2 shows the flow from the abstracts to gene categorization. After filtering the results to consider only human genes, the list was reduced to 253 genes. These genes were sorted using the number of associated abstracts. There was a clear distinction of genes depending on their corresponding number of abstracts. The well-known PAH-susceptible genes were mentioned in more than 10 abstracts; 13 genes had 10 or more occurrences in the abstracts and some of these genes were clearly recognized for their biological functions in PAH. These genes included a *BMPR2*, *ACVRL1* (known also as *ALK1*, activin receptor-like kinase 1), *ENG* (endoglin), and those of the SMAD family. We did not find mutations in *SMAD1*, *BMP4*, *BMP2*, *ID1*, or *SMAD5*, which had 19, 16, 15, 11, and 11 associated abstracts, respectively. In addition, the phenylalanine hydroxylase gene, whose official gene symbol is PAH, was annotated in all abstracts as a gene and as a disease, it was therefore classified as an annotation error. After the revision, from the 15 genes that were found in 10 or more abstracts, five of these genes (*BMPR2*, *ACVRL1*, *ENG*, *EDN1*, and *SMAD9*) were found to have some evidence of mutations in PAH, one gene (*NOS3*) was found to be mutated in a related disease, and one gene, serotonin transporter (*SLC6A4*), was classified as negative evidence.

For the 222 genes that appeared in fewer than 9 abstracts, a full abstract revision was performed and a full-text read was done if the abstract was unclear. From these 222 genes, 16 genes were found to have evidence of mutations. Thus, a total of 21 genes were found to have evidence of mutations. Table 2 provides the complete list of genes.

**Table 2:** The 21 genes having genetic evidence of mutations

Symbol	Name	Abstracts	Kits
ACVRL1	Activin A receptor like type 1	30	10
AGTR1	Angiotensin II receptor, type 1	3	-
BMPR1B	Bone morphogenetic protein receptor, type 1B	5	2
BMPR2	Bone morphogenetic protein receptor, type II	177	12
CAV1	Caveolin 1	6	9
EDN1	Endothelin 1	19	-
EDNRA	Endothelin receptor type A	1	-
EIF2AK4	Eukaryotic translation initiation factor 2 alpha kinase 4	5	1
ENG	Endoglin	24	10
KCNA5	Potassium voltage-gated channel subfamily A member 5 member 5	7	2
KCNK3	Potassium two pore domain channel subfamily K member 3	5	5
NOS2	Nitric oxide synthase 2	2	-
NOTCH3	Notch 3	1	1
SERPINE1	Serpin family E member 1	3	-
SIRT3	Sirtuin 3	1	-
SMAD4	Smad family member 4	9	2
SMAD9	Smad family member 9	16	9
TBX4	T-box 4	1	-
THBS1	Thrombospondin 1	1	-
TOPBP1	Topoisomerase (DNA) II binding protein 1	1	-
TRPC6	Transient receptor potential cation channel, subfamily C, member 6	3	-

Interestingly, of these 21 genes, 10 have been poorly studied (six appeared in only one related abstract, one gene appeared in two abstracts, and three genes appeared in three abstracts). For example, angiotensin II receptor type 1 (AGTR1), a potent vasopressor hormone and an important regulator of cardiovascular hemodynamics [15], appeared in two publications in 2008 and was not included in any PAH gene panel despite being directly related to PAH hypertension. More importantly, endothelin 1 (EDN1), a potent vasoconstrictor [16], was mentioned in 20 abstracts but was not included in any of the PAH panels that we considered in this study. In addition to the above genes, *EDNRA*, *NOS2*, *SERPINE1*, *SIRT3*, *TRPC6, TBX4, THBS1,* and *TOPBP1* were not included in any of the reviewed genetic panels (see the column Kits in Table 2).

We also found nine genes that had other genetic evidence in PAH (*CBLN2, CYP1B1, GNB3, HLA-DQB1, HLA-DRB1, HTR2B, PTGIS, SOD2,* and *TGFB1*). Two of the genes were related to the major histocompatibility complex [17], while the others were related to locus markers instead of specific mutations [18–20], synergistic mutations with other PAH-related genes [21], treatment efficiency [22], or epigenetic alterations [23].

From the 222 genes, 83 genes were found to be biologically related to PAH without definitive evidence of mutations. This suggested that the molecular mechanism implicated in PAH was more complex than was previously thought [7]. Many of these genes were either associated with changes in gene expression levels, found in experiments performed in cells from patients or animal models carrying a mutation in hallmark genes, or expressed in response to treatment studies.

Of the 222 genes, 41 were found to carry mutations or other genetic alterations in patients with other diseases in which PAH was mentioned as a related disease or as another classification of arterial hypertension. Some examples included chronic obstructive pulmonary disease, systemic sclerosis, hepato-pulmonary syndrome, and pulmonary arterial hypertension of the newborn. Additionally, some of the 41 genes were mentioned in abstracts because they were used as markers in cell lineages, cell lines, or activation or deactivation pathways, or they were used to test for changes in expression, but not mutations were tested in PAH patients.

Thirteen genes found in people with PAH had no significant polymorphism or associated mutations. Another 45 genes were wrongly annotated; these genes had “*open reading frame”* generic names that were confused with “OR” (odd ratios) co-occurring with PAH terms or names that were confused with the acronyms of diseases, methods, or chemicals.

The classification of all genes can be found as Additional file 1.

### Location of mutations in identified PAH genes

**Fig. 3:**
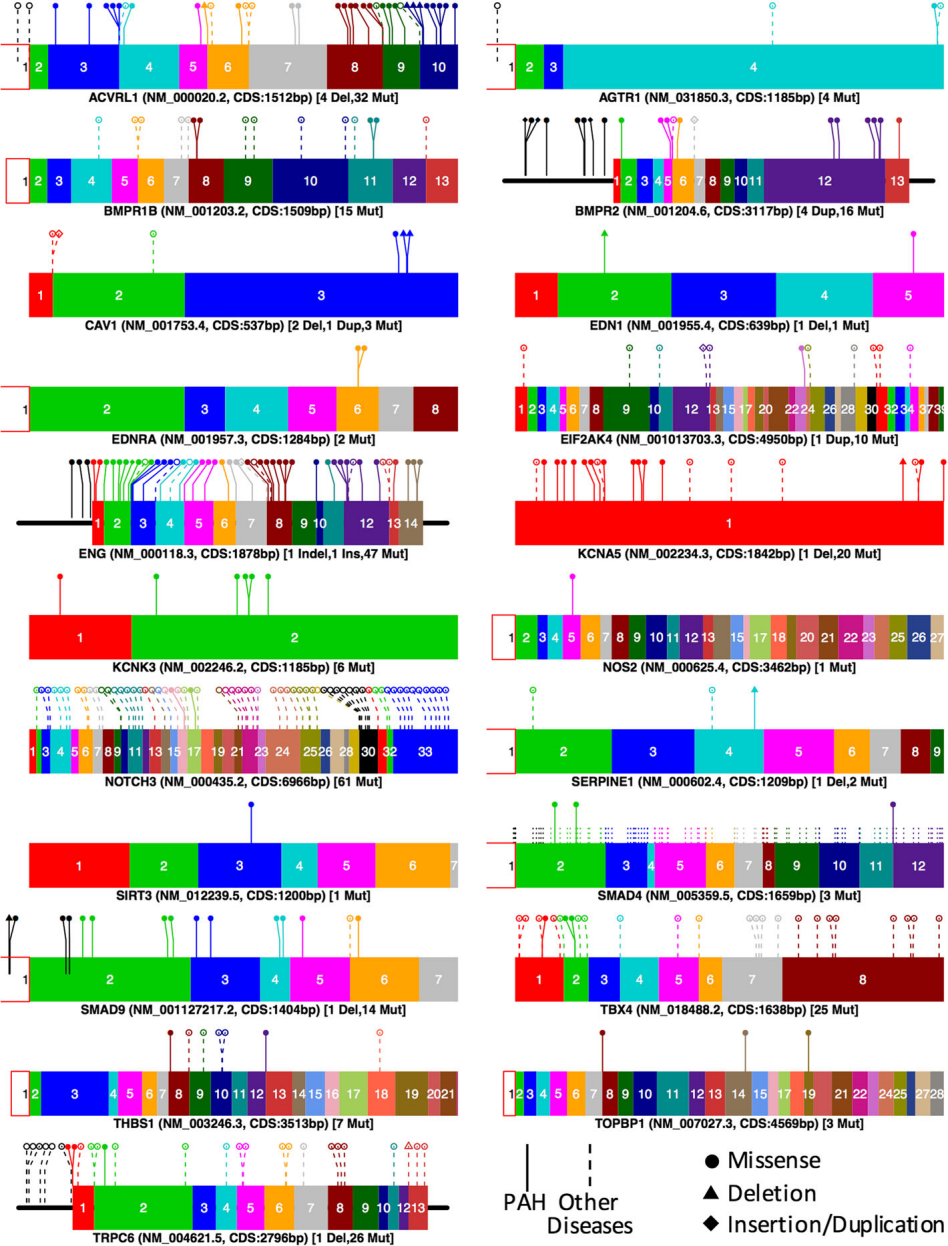
Mutations reported and curated from the 21 genes having evidences of mutations. Only exons are shown and numbered except in cases of mutations upstream of the first exon. Color figure can be viewed in the online issue

To provide an overview of the location of the mutated regions for planning genetic testing strategies, we collected all the mutations as reported in ClinVar that were related to single nucleotide variants, small deletions, and insertions for the 21 genes identified with specific mutations. For genes with mutations that were not registered in ClinVar, we obtained the mutations from original publications: 64 additional mutations in 19 genes. The complete list of mutations is provided in Additional file 1. The map of these mutations within the gene model is shown in Fig. 3 Most of the mutations were missense single nucleotide mutations, frameshift mutations (deletions or insertions), and nonsense mutations (stop codon gains). Most of the mutations were found in coding regions. Many of the mutations specific for PAH are surrounded by mutations that have been found in other diseases. From the number of marked mutations, it is clear that some genes have been poorly studied.

### Pathway analysis of identified PAH genes

**Fig. 4:**
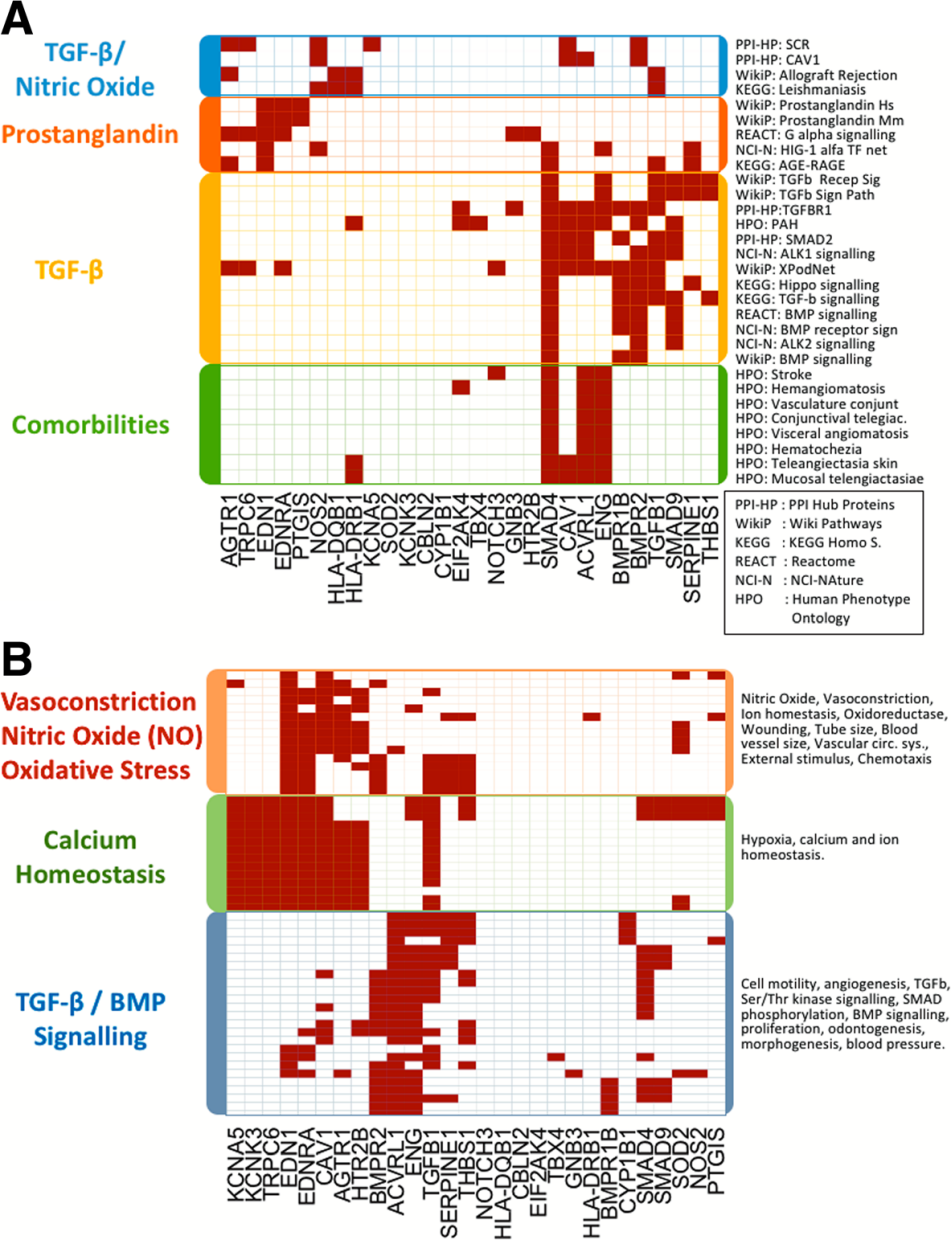
Functional analysis of the genes found genetically altered in PAH. **a** Pathways and comorbilities. **b** Gene ontology terms. Only the genes with annotations in these terms are included in the mapping. Color figure can be viewed in the online issue

We performed a composed functional analysis of genes with experimental evidence of mutations and other evidence of mutations for PAH to appreciate the different correlated genes and the altered signaling pathways that occurred from these mutations; the consequences of the mutations were considered at a phenotypical level (Fig. 4a). For this analysis, we used DAVID [13] and EnrichR [14] as described in the methods section. It is well-known that either primary PAH, familial PAH, or secondary pulmonary hypertension are characterized by mutations in the *BMPR2* gene and along the TGFβ signaling pathway, which include *SMAD4*, *SMAD9*, *CAV1*, and *ENG*. This was confirmed in our analysis. However, we also found possible connections with the prostaglandin signaling, nitric oxide, and calcium homeostasis, which are discussed below.

## Discussion

Heterozygous BMPR2 mutations account for approximately 75% of hereditable PAH and up to 25% of presumably sporadic PAH cases [5]. The most important registries around the world have identified an incidence of less than 5% for hereditable PAH with a penetrance range between 20 and 80%. The REVEAL registry recognized an incidence of 2.7% [24], while the French registry identified 3.9% [25]. However, the incidence in other registries from China [26] and Brazil [27] is unknown. Recent guidelines of the European Cardiology Society [2] recommend analyses for genetic alterations in *BMPR2*, *BMPR1B*, *EIF2AK4*, *CAV1*, *KCNK3*, and *ENG* where *BMPR2* has a key role in the etiological factor of the disease. Currently, genetic testing is a suggestion, but not a recommendation because of the level of evidence that supports the use of genetic testing in the diagnostic approach. In addition, genetic counseling is an issue that must be analyzed from an ethical perspective to allow the patient to make appropriate decisions.

Through PubMed and Internet searches, we found 12 available kits to identify genetic alterations related to PAH. Some of these kits are also applied to diverse cardiovascular disorders, such as dilated cardiomyopathy, arteriovenous malformations, and congenital defects. On average, these kits considered around eight genes, which were compared with the 21 genes identified (see column Kits in Table 2). Although one kit considered 11 genes of the 21 genes, most kits considered on average only 5 genes (*BMPR2*, *ACVRL1*, *ENG*, *CAV1*, and *SMAD4*). None of the kits included genes that have been associated with PAH, such as *AGTR1*, *TBX4*, *EDN1*, *EDNRA*, *NOS2*, *SERPINE*, *SIRT3*, *THBS1*, *TOPBP1*, or *TRCP6*. For example, *AGTR1* has been related to later age PAH at diagnosis [28], *TBX4* has been related to childhood-onset of PAH [29], and *EDN1*, *EDNRA*, and *NOS2* have been related to susceptibility to develop the disease and its clinical course as well as susceptibility to systemic sclerosis-related PAH [16, 30–32]. *TOPBP1* and *TRCP6* have been linked to cellular protection and proliferation, respectively [5, 33–35]. *SERPINE1* has been linked with a risk of venous thromboembolism [36], while *SIRT3* was related to mitochondrial dysfunction [37]. *THBS1* has been identified [38], but not studied functionally. The identification of an important number of genetic alterations offers the potential to expand our knowledge about PAH. This provides a better understanding of the onset and progression of the disease, as well as the development of biomarkers for early diagnosis and stratification and potential therapeutic strategies, improving the clinical scenario for patients [5].

The map of mutations (Fig. 3) suggests that mutations specific for PAH (continuous line) are widely distributed in a few genes: *ACVRL1*, *BMPR2*, *EIF2AK4*, *ENG*, *KCNA5*, and *SMAD4*. Nevertheless, there are many other mutations not yet found in PAH patients that have been found in other diseases. It is sensible to believe that mutations that affect other PAH-related disease genes are likely to have an effect in PAH. Consequently, most of the genes are affected along the whole coding sequence, such as *AGTR1*, *CAV1*, *EDN1*, *NOTCH3*, *SMAD9*, *TBX4*, and *TRPC6*. For all these genes, testing specific mutations or sequencing selected regions does not seem to be a good strategy, instead, a more broad strategy, such as sequencing, would be needed. Figure 3 also shows that *ACVRL1*, *ENG*, *SMAD4*, *SMAD9*, and *TRPC6* have some mutations in the 5’UTR suggesting that coding mutations are not the only mutations that affect PAH. This concur with recent reports that found mutation in the promoter of *BMPR2* [39]. These mutations may alter the binding of transcription factors or the transcriptional machinery affecting the gene levels instead of the function of the produced protein. Therefore it is important to test this types of alterations.

Around 300 different mutations in *BMPR2* have been studied and found to be related to a PAH diagnosis [6, 40]. BMPR2 is a member of the TGFβ receptors superfamily, which consists in an extracellular and a transmembrane motif and kinase domains. Recent research showed that mutations in BMPR2 promote cell division and prevent cell death, resulting in an overgrowth of cells in small arteries throughout the lungs [41]. As reviewed here, mutations in other genes also contribute. In this context, our functional analysis showed that the mutations were, collectively, related to other biological processes, diseases, and comorbidities, including hypoxia, pulmonary hypertension, hemangiomatosis, and abnormality of the vasculature of the conjunctiva (Fig. 4). Other functional associations found were related for example to stroke, or telangiectasia. It should be determined if these vascular disorders are directly related to PAH.

Mutations associated with the genes in the TGFβ signaling pathway, such as *ALK2* (*ACVR1*), *ALK1* (*ACVRL1)*, and *CAV1*, are also very important. ALK1 and ALK2 function to activate other proteins and genes in the TGFβ and BMPR2 pathways [42]. *CAV1* has been extensively reported with many confirmed mutations specific for PAH [43, 44]. This gene codes a protein that contains 178 amino acids in its α-isoform. The expression of this protein is implicated in caveolae formation, which are plasma membrane structures that are specialized microdomains known as lipidic rafts. Caveolae are rich in superficial receptors that interact with cell membranes. These receptors are essential in order to activate the signaling pathways. The TGFβ and the nitric oxide signaling pathway are targeted by the plasmalemmal caveolae in endothelial cells; these pathways are involved in PAH development. For instance, CAV1 can modify the signal of TGFβ at the plasmatic membrane level. Thus, mutations in *CAV1* could be related to *BMPR2* mutations in PAH [43, 45]. During our analysis, we appreciated how a huge number of these genes and genes that were associated with the TGFβ signaling pathway were related to the vasoconstriction processes. It is also possible to appreciate how some of these genes are related to the synthesis of prostaglandins (Fig. 4a); these genes relate to vasoconstriction and increase tissue permeability. In this regard, many different drugs have been developed for vasodilatation of the pulmonary vasculature. One interesting example is sildenafil, which inhibits phosphodiesterase and enhances the vasodilatory effects of nitric oxide in PAH to promote relaxation of the vascular smooth muscle and increase blood flow. This drug produces a relatively selective reduction in pulmonary artery pressure without adverse systemic hemodynamic effects and is one of the most widely used drugs in treating PAH [46, 47].

Finally, genes related to redox homeostasis and calcium handling were also classified. In the analysis, genes with specific mutations for PAH, such as *CAV1*, *AGTR1*, *EDN1*, and *KCNA5*, were related to the regulation of homeostasis and ion controls. As a consequence, one of the pathways shown in the analysis was for the regulation of cytosolic calcium; many of the altered genes in PAH code for the transmembrane proteins that are responsible for calcium transport. As shown in Fig. 4b, the regulation of homeostasis and ion control within the cell are all controlled by the same genes. It is important to mention that these genes, such as *EDN1*, *EDNRA*, and *SMAD9*, are also associated with the nitric oxide biosynthesis process. For example, EDN1 and EDNRA are that are common in homeostasis control in the cell or in nitric oxide biosynthesis processes. Besides, these genes are associated with oxidative processes; for example, EDNRA, EDN1, and CAV1 are related to the synthesis of different oxidoreductases that control oxidative stress within cells. One of the most novel genes associated with oxidative stress is *SIRTUIN3*, which protein is involved in the post-translational modification of several proteins complexes involved in the antioxidant cellular and mitochondrial system [48]. Surprisingly, this gene does not appear in the results of our analysis in any of the different pathways. However, the importance of the mutations in this gene in PAH has been clearly documented [37].

The results of our systematic review propose that other genes whose prevalence or incidence have not widely studied may be important in the pathogenesis of PAH. In particular, the pathway analysis and the biological function of genes support our view. For research, it may be sensible to assess all genes. For clinical purposes, or if cost is an issue, a series of panels may be used; the first including well-known and more likely mutated genes like *BMPR2*, *ACVRL1*, *ENG*, *EDN1*, *SMAD9*; then, if no mutations are found, *SMAD4*, *KCNA5*, *CAV1*, *BMPR1B*, *EIF2AK4*, and *KCNK3* may follow; and so on with the remaining genes.

The limitations of this study relate to the PAH classification, which has recently been modified; several reports could be potentially included in our analysis that used previous classifications of PAH. Based on the results of our systematic approach, we explored the relative risk associated with mutations and the occurrence of PAH. However, because of the heterogeneity of reports, populations, and some case reports, the associations were sometimes difficult and confusing to determine. Few studies reported associations between genetic alterations and the manifestation of PAH. We were also limited by the abstracts annotations provided by third party tools like PubTator [11] where, overall, we observed accurate annotations but also some mistakes and time delays in the annotations.

## Conclusion

In summary, the gene list we provided, in combination with recent sequencing methods and larger studies, may contribute to accurately estimate the relative genetic incidence and risk of PAH. This would expand the knowledge of PAH in terms of genetic counseling, early diagnosis, incidence of mutations, clinical assessment, therapeutic approach, and prognosis of the disease. A future study could validate this gene-targeted panel test in PAH patients.

## Additional file

Additional file 1: “Table S1-Mutations Per Gene.xlsx” contains the list of curated mutations including those not present in ClinVar. (XLSX 92 kb)

## Abbreviations

GO: Gene Ontology
PAH: Pulmonary arterial hypertension
